# Intra-articular injection of triamcinolone acetonide releasing biomaterial microspheres inhibits pain and inflammation in an acute arthritis model

**DOI:** 10.1080/10717544.2019.1568625

**Published:** 2019-03-07

**Authors:** Imke Rudnik-Jansen, Karin Schrijver, Nina Woike, Anna Tellegen, Sabine Versteeg, Pieter Emans, George Mihov, Jens Thies, Niels Eijkelkamp, Marianna Tryfonidou, Laura Creemers

**Affiliations:** a Department of Orthopedics, University Medical Center Utrecht, Utrecht, the Netherlands;; b DSM Biomedical B.V, Geleen, The Netherlands;; c Department of Clinical Sciences of Companion Animals, Utrecht University, Utrecht, the Netherlands;; d Laboratory of Translational Immunology, University Medical Center Utrecht, Utrecht University, Utrecht, the Netherlands;; e Department of Orthopedics, Maastricht University Medical Center Utrecht, Utrecht, the Netherlands

**Keywords:** Arthritis, microspheres, polyesteramide, polylactic-co-glycolic acid, synovitis, triamcinolone acetonide

## Abstract

Inflammation of the synovium and joint capsule is a main driver of pain in an osteoarthritic (OA) joint. Triamcinolone acetonide (TAA) is a classical corticosteroid that reduces synovitis and alleviates pain, albeit transiently. Biomaterial-based local TAA release may prolong the suppression of pain without the need for multiple injections. Polylactic-co-glycolic acid (PLGA) formulations of TAA prolong OA pain relief to a limited extent. A novel polyesteramide (PEA) microsphere platform allows for extended release in the OA joint for over 3 months. To evaluate their effect on pain and inflammation, TAA-loaded microspheres were intra-articularly delivered to the knee joint in a rat model of acute arthritis induced by intra-articular injection of streptococcal cell wall peptidoglycan-polysaccharide (PGPS) and subsequent flare-ups by intravenous PGPS injections. PEA-loaded microspheres were benchmarked with TAA-loaded PLGA microspheres and bolus TAA injection. TAA treatments were injected intra-articularly before the first induced flare-up. TAA-loaded PEA and PLGA microspheres reduced joint swelling and signs of pain-like behavior over the entire study period, as assessed by weight bearing and referred mechanical hypersensitivity, whereas bolus suspension was effective for a shorter time period. TAA-loaded PEA microspheres reduced lameness to a greater extent than TAA-loaded PLGA microspheres. In conclusion, a single intra-articular injection of TAA-loaded PEA microspheres reduced joint swelling and induced longer pain relief compared to bolus injection. Hence relief of inflammation and pain by PEA-based delivery of TAA may prove to be effective and durable.

## Introduction

Osteoarthritis (OA) is the most common form of arthritis, affecting people worldwide, with rising prevalence (March et al., 2014). This degenerative joint disease is characterized by cartilage breakdown, fibrotic changes to the joint capsule, bony changes, and inflammation of the synovial membrane (Samuels et al., 2008). These phenomena result in pain and reduced mobility, thereby negatively affecting the quality of life in OA patients. An important feature and source of pain in both OA and many other joint pathologies is synovial inflammation, or synovitis. Synovitis causes joint pain (Hill et al., 2007; Ishijima et al., 2011) and is characterized by synovial hypertrophy, increased neovascularization and influx of immune cell infiltrates that secrete pro-inflammatory mediators (Smith et al., 1997; Krenn et al., 2006). In addition to triggering several clinical signs, it can extend cartilage breakdown (Sellam & Berenbaum, 2010). Targeting synovial inflammation is, therefore, an important treatment strategy in OA patients.

For decades, corticosteroids have been widely used to alleviate pain symptoms in various inflammatory diseases (Hayashi et al., 2008; Li et al., 2011; Laste et al., 2013). Triamcinolone acetonide (TAA) is a corticosteroid that is intra-articularly (IA) injected to reduce OA- and/or mono-articular rheumatoid arthritis-related pain (Makrygiannakis et al., 2012; van Middelkoop et al., 2016; Kumar et al., 2017). Although IA TAA provides analgesia, it only lasts relatively short with a maximum of up to 8 weeks (Bellamy et al., 2006). Multiple TAA injections or long-term systemic use might increase the risk of infection or entail risks of overdosing inducing side-effects (Huscher et al., 2009; Xing et al., 2014). Local sustained release may overcome these disadvantages. Recently a microsphere formulation of TAA in polylactic-co-glycolic acid (PLGA), a commonly used FDA-approved biomaterial, was launched to inhibit pain and inflammation for prolonged periods in OA knee joints (Bodick et al., 2015; Kraus et al., 2018). Although TAA release by these PLGA microparticles initially reduced pain to a higher extent than the common bolus formulation, the duration of action was not prolonged compared to bolus injection, possibly due to the generally fast degradation rate of PLGA (Xu et al., 2017). As such, there is still an unmet clinical need for extended-release formulations of TAA. A recently developed polyesteramide (PEA) microsphere platform based on natural α-amino acids was shown to be retained in the OA-knee joint for at least 12 weeks, suggesting its potency towards prolonged delivery (Janssen et al., 2016). Similarly to PLGA, the foreign body responses *in vivo* to PEA is very mild, with limited cell infiltrate or tissue damage, also in the joint cavity (Janssen et al., 2016; Peters et al., 2017). When loaded with the anti-inflammatory drug celecoxib, 16 weeks after IA injection in surgically induced OA joints, inhibition of inflammation and the OA bone phenotype was demonstrated (Tellegen et al., 2018). Also loaded with TAA, intra-articular injection of PEA microspheres reduced the mild synovial inflammation of a collagenase-induced OA rat model during the 7 weeks follow up (Rudnik-Jansen et al., 2017). However, in both studies, the pain was difficult to assess and hence no analgesic effects of the delivered anti-inflammatory drugs could be measured. Typically, the inflammatory responses in most OA animal models (Lampropoulou-Adamidou et al., 2014) are limited. Moreover, these models are commonly based on prey animals that usually hide symptoms of pain (Mayer, 2007), hence in general detection of analgesic effects of novel treatments is hampered in these models.

Therefore, to gain insight into the capacity of TAA-loaded PEA microspheres to generate prolonged relief of joint pain, a rat model of acute inflammatory arthritis was used. PEA microspheres loaded with TAA were intra-articularly delivered and benchmarked to TAA released from PLGA microspheres, and to the bolus TAA formulation available as a medical product. It is unlikely that the PEA polymer will induce inflammatory responses by itself. To evaluate inflammatory effects and pain-like behaviors in the general acute arthritis model, empty PEA-treated animals were included.

## Material and methods

TAA bolus injections consisted of TAA suspension (40 mg/mL, Kenacort; Bristol-Myers Squibb, Woerden, The Netherlands) diluted in 0.9% sterile saline to obtain required TAA dosage. Streptococcal cell wall peptidoglycan-polysaccharide (PGPS; 100 P fraction with 5 mg rhamnose/mL PGPS from Lee Laboratories) was used to induce unilateral synovitis and flare-ups in the experimental knee joint.

### Synthesis of polylactic-co-glycolic acid (PLGA) and polyesteramide (PEA) microspheres

PEA was synthesized according to a previously published method (Tsitlanadze et al., 2004; Tellegen et al., 2018). PEA polymer was dissolved in dichloromethane (DCM Merck Millipore, Darmstadt, Germany) to obtain unloaded microspheres, or 30 wt% TAA was dispersed in the polymer/solvent mixture. The suspension was sonicated in a water bath for 3 min. Then the formulation was emulsified in 20 mL of water phase, (poly(vinyl alcohol, Sigma-Aldrich, Darmstadt, Germany. 1 wt% and NaCl 2.5 wt%) by the use of an Ultra-Turrax, and stirred at 8000 rpm for 3 min. PLGA microspheres loaded with TAA were synthesized using solid oil-in-water emulsification technique. PLGA (Resomer^®^ RG 753 H, Evonik, Darmstadt, Germany) was dissolved in DCM (Merck Millipore, Darmstadt, Germany). TAA (25 wt%) was dispersed in the polymer/solvent solution and sonicated in a water bath for 3 min to obtain the oil phase. The latter was emulsified in 20 mL of water phase, (poly(vinyl alcohol, Sigma-Aldrich, 1 wt% and NaCl 2.5 wt%) by the use of an Ultra-Turrax, and stirred at 4000 rpm for 3 min. After emulsification of the PLGA and PEA particles, microspheres were let to harden overnight into a hardening bath of 100 mL water phase under air flow. Particles were cooled with an ice-bath for 1 h and thereafter washed with 0.04% Tween 80. Excessive surfactant was removed by centrifugation. Before freeze-drying to remove residual surfactant, particles were resuspended in 0.04% Tween 80. Once dried, closed vials were sterilized with ɣ-radiation on dry ice.

### Characterization of microspheres

Size distribution of the PLGA and PEA microspheres was measured by static light scattering using a Malvern Mastersizer 2000S (Sysmex Netherlands B. V. Etten Leur, the Netherlands). To determine loading, 10 mg of particles were weighed and dissolved in methanol for PEA microspheres or acetone for PLGA microspheres. The solutions were diluted with PBS in such a way to fit within the calibration line. *In vitro* release of TAA from PLGA- and PEA-loaded microspheres was followed for 42 days. Approximately 10 mg of particles were weighed in 50 mL glass centrifuge tubes and 40 mL of PBS were added. The vials were placed on an IKA shaker at 37 °C and 100 rpm. At specific time points, 1 mL supernatant was collected for HPLC analysis; first vials were centrifuged for 1 min at 2000 rpm, 36 ml supernatant was removed and refreshed with the same amount of PBS buffer. Samples were analyzed using a Waters HPLC system (Waters, Milford, MA, USA).

### Study design

**Figure 1.**
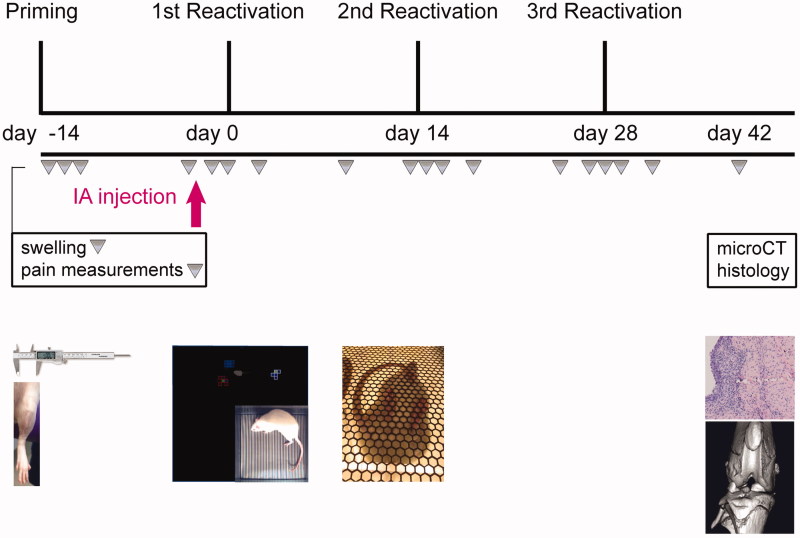
Schematic overview of the study design. First, local synovitis was induced by priming the experimental knee joint for PGPS by intra-articular (IA) injection of 0.17 mg/mL PGPS (day-14). The next days, local inflammation and pain were evaluated. To reactivate flare-up episodes of synovitis, PGPS was administered by IV injections of 0.28 mg/mL PGPS (day 0, 14, 28). Treatments were administered by single IA injections in the experimental joint 2.5 h before first reactivation (day 0), indicated by the pink arrow. Inflammation, lameness, and pain were measured once every 0, 1, 2, 4, and 10th day after PGPS administration. At day 42, rats were culled, scanned with micro-CT, and joints were harvested for histological analyses.

Twenty-four 8 weeks old female Sprague-Dawley rats (Charles-River laboratories, The Netherlands) were used in the study. Rats were allowed to acclimatize for one week and housed in groups (3–4 rats, randomized for treatment) in polycarbonate cages with wire tops, wood chip bedding, and access to *ad libitum* food and tap water. Local synovitis was induced by priming the left joint (e.g. experimental joint) for PGPS by IA injection of PGPS (25 µL PGPS of 0.17 mg/mL) under general isoflurane anesthesia (day-14). The next day, animals were evaluated for local inflammation by measuring joint thickness, and pain measurements were performed. One animal characterized as non-responder (Esser et al., 1985), was removed from the study. Synovitis symptoms reduced over time and on day 0, 14, and 28 flare-up episodes of synovitis were reactivated in the experimental knee joints by intravenous injections of 0.5 mL PGPS via the tail vein (0.28 mg/mL PGPS). Two and half hours before the first reactivation (day 0), animals were randomly divided into the following treatment groups; TAA bolus suspension (*n* = 6), PEA microparticles loaded with TAA (*n* = 6) or PLGA microparticles loaded with TAA (*n* = 6), and control group injected with empty PEA microparticles (*n* = 6). Treatments were administered by single IA injections in the experimental joint. The TAA dosage in every experimental group was 2.5 mg/mL TAA in 25 µL, equivalent to 12.5 mg TAA in humans, based on body weight. The experimental unit in this study was a single animal and the analysis unit was the outcome of a single animal over a follow up starting from treatment (day 0) until termination (day 42). Primary experimental outcomes included joint swelling, lameness, mechanical hypersensitivity, and asymmetry in weight bearance during the complete follow-up. Joint swelling as an indication of inflammation was measured every 0, 1, 2, 4. and 10th day after PGPS administration. Pain measurements using a lameness score (yes/no), von Frey were done every 0, 1, 2, 4, and 10th day after PGPS administration. Dynamic weight bearing assays were done every 2 and 10th day after PGPS administration. Forty-two days after TAA delivery, animals were terminated by CO_2_ asphyxia, subsequently scanned with micro-CT and hind knee joints were harvested for histological processing and analyses. An overview of the study set up is provided in Figure 1. Animals that showed lameness in combination with swelling of both hind paws were given additional pain medication (s.c. carprofen, 5 mg/kg) on that day. The study design and protocol (AVD108002015282, WP#800-15-282-01-003) were approved by the national central commission of animal experiments and experiment were performed according to DIRECTIVE 86/609/EEC and FELASA guidelines.

#### Behavioral assays

All injections (IR) and behavioral assays (IR/KS) were performed and analyzed in a randomized fashion by observers blinded to treatment.

#### Swelling

Swelling was evaluated by measuring knee joint thickness in mm using a digital caliper. For each time point, joints were measured 3 consecutive times, averaged and recorded as one data point. Swelling was calculated by subtracting baseline measurements (day-14) from the actual time point measurement to indicate acute inflammation evoked by PGPS injections.

#### Referred mechanical hypersensitivity

Mechanical hypersensitivity provoked by joint inflammation was assessed by applying von Frey hairs to the hind paw (Chaplan et al., 1994) and 50% threshold was determined using the up-down method (Minett et al., 2014). The up-down von Frey method was used to determine the mechanical force required to elicit a paw withdrawal response in 50% of animals. Prior to the assessment, rats were placed in a plexiglass test cage with a wire mesh floor and allowed to acclimate for 10 minutes. In case of animals displayed the following behaviors (curling toes, eversion of the paw, and non-weight bearing) continuously for at least 5 min, the lowest value of the von Frey hair was used (50% threshold −0.6 g).

#### Visible lameness

Lameness was used as a measure for the extent of restriction in the experimental joint by inflammation and pain. Lameness was evaluated (yes/no) in the plexiglass test cage with a wire mesh floor and scored as such if animals did not load the affected hind paw during ambulation for 10 minutes or more. An example is shown in Supplementary Video 1.

#### Dynamic weight bearing analysis

Weight-bearing symmetry and paw surface areas were measured and analyzed using the advanced dynamic weight bearing (DWB) (Bioseb, module version 1.4.2.98; Boulogne, France). The weight on each separate paw (g), surface area of each separate paw (mm^2^) and weight on other weight-bearing parts (g; front paw, contralateral paw, tail) was measured and the following ratios were determined; weight on ipsilateral hind paw/weight on contralateral hind paw, weight on ipsilateral hind paw/weight on all other weight-bearing body parts. Asymmetrical weight bearing indicated a painful affected experimental joint (Prado et al., 2018; Willemen et al., 2018). Moreover, the surface distribution of the hind paw of the affected leg was determined (mm), because during painful conditions in the paw these are reduced (Tetreault et al., 2011). For each testing period, each rat was individually placed in a 22 cm × 22 cm × 30 cm plexiglass chamber with floor sensors composed of 44 × 44 captors (10.89 mm^2^ per captor). Rats were allowed to move freely and explore for 10 sec prior to data collection. Then, a high resolution 640 × 480 USB-based camera recorded each movement for 5 min and the following parameters were measured: weight on each separate paw (g), surface area of each separate paw (mm^2^) and weight on other weight-bearing parts (g; front paw, contralateral paw, tail). Three measurements on different days were averaged and used as baseline, thereafter each rat was measured once per time point. A blinded observer (IR) validated all data by comparing live recordings with a scaled map of the activated sensors to verify the body part placements of the animal. Time spend rearing or washing was excluded from the analyses and a minimum of 1 min of validated testing period was used to calculate mean values. The DWB software (Bioseb, DWB software v1.3) determined surface and pressure parameters automatically. Zone parameters were set for the analysis as followed: low weight threshold ≥1 g, weight threshold ≥2 g, surface threshold of 3 (in order to be consider a valid zone). For each time segment that was stable for more than one second, zones that met the above criteria were validated and assigned as either right or left, and front or hind. A mean value for the weight and area of each zone was calculated over the entire testing period, based on the length of time of each validated segment.

At one single time point, one animal (group PLGA microparticles loaded with TAA at day 30) did not load any of his limbs. Therefore for this data point, the highest score in the PLGA group was taken for every single DWB read-out parameter.

### Micro-CT analyses

Directly after CO_2_/O_2_ asphyxiation, animals were imaged using a Quantum FX µ-CT scanner (PerkinElmer, Waltham, MA) with parameters time = 3 min, isotropic voxel size = 30 µm^3^, tube voltage of 90 kV, tube current = 180 µA. Serial 2 D images were reconstructed from the obtained 3 D reconstructed images using software Analyze 11.0 (PerkinElmer, Waltham, MA, USA). ImageJ software (ImageJ, 1.47v, NIH, Bethesda, USA) was used for all analyses. Serial 2 D scans of the femur, tibia, and patella were evaluated for subchondral sclerosis, osteophytes, bone cysts and loose bodies according to a multi-modality scoring system for rats evaluated previously (Panahifar et al., 2014).

### Histology

After micro-CT imaging, all hind limbs were collected for histological analysis and joints were fixed in 4% formaldehyde solution (Klinipath BV, Netherlands) at room temperature for 1 week. Thereafter, joints were decalcified at room temperature in 0.5 M EDTA (VWR international BV) solution for a total of 8 weeks, re-fixating for 3 days in 4% formaldehyde solution every 2 weeks, and embedded in paraffin. Five micrometer transversal knee joint sections were cut and stained either with Safranin-O/Fast green or hematoxylin/eosin to evaluate cartilage degeneration using the Mankin score (Mankin & Lippiello, 1970) (0 complete healthy – 14 total joint destruction) or synovitis using the Krenn score (Krenn et al., 2006) (0 healthy – 9 severe synovitis), respectively. Scoring was done at random by two blinded observers independently and scores were averaged (AT and IR).

### Statistical analyses

Data were analyzed using IBM® SPSS® Statistics version 21 (IBM Corporation, Amsterdam, the Netherlands). For each dataset, the course over time (day 0–day 42) for every animal was used as an experimental unit and statistical analysis was performed to detect differences between all groups; TAA bolus suspension (*n* = 6), PEA microparticles loaded with TAA (*n* = 5) or PLGA microparticles loaded with TAA (*n* = 6) and control group injected with empty PEA microparticles (*n* = 6). Observations were independent and statistical significant differences between different time points were not evaluated in this study. Equality of data variances was evaluated by Q-Q plots and homoscedasticity of residuals by scatterplots. In case ANOVA assumptions were not met, the Kruskal–Wallis test was used to analyze nonparametric data. The course over time of joint swelling, 50% response thresholds, and weight asymmetry, as ratio ipsilateral/contralateral and loading surface ipsilateral paw, were analyzed by nonparametric Kruskal–Wallis test with *post-hoc* pairwise comparisons using the Dunn-Bonferroni approach. Weight-bearing ratio of ipsilateral compared to all other weight-bearing parts and surface area of ipsilateral compared to all other surface area parts was analyzed with randomized block design ANOVA to correct for donor variability. If statistical significant differences were found between groups, Sidak *post hoc* analysis was used to correct for multiple comparisons. Lameness incidence was added up and cumulative data were analyzed with contingency tables followed by chi-square *post-hoc* tests based on adjusted standardized residuals with Bonferroni correction for multiple comparisons. *p* < .05 was taken to indicate statistical significance. Corrections regarding the multiplicity issue were done to correct for multiple comparisons, rather than corrections for multiple testing.

## Results

### Microsphere characterization

The size distribution of all three microsphere batches was determined. All batches showed a monomodal distribution, with a size distribution ranging from 8 to 50 µm. Mean particle size of empty PEA microspheres was 38.2 µm with a polydispersity index (PDI) of 1.75; PLGA microspheres 39.4 µm with PDI of 1.30 and PEA microspheres 23.8 µm, with PDI of 1.30. TAA loading of PLGA-loaded microspheres resulted in 23 wt% TAA and for PEA-loaded microspheres in 28 wt% TAA. For both formulations, the loading efficacy was 92%.

**Figure 2.**
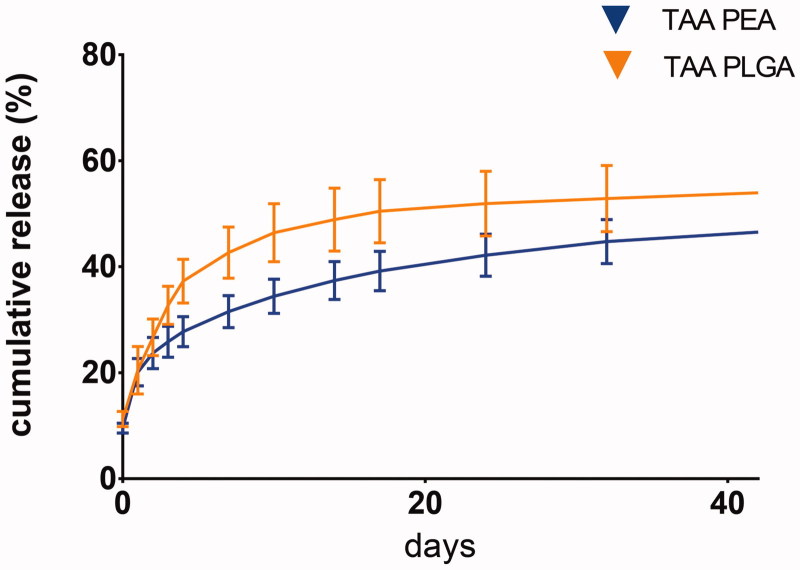
Cumulative TAA release from PLGA and PEA microspheres in PBS buffer. The concentration of TAA release in PBS medium as determined by HPLC throughout 42 days. For each polymer, 3 batches were used as technical replicates.

*In vitro* TAA release in PBS buffer showed an initial burst at day one with a gradual increase of TAA release thereafter, for both microsphere platforms (Figure 2).

### General observations acute arthritis model

**Figure 3.**
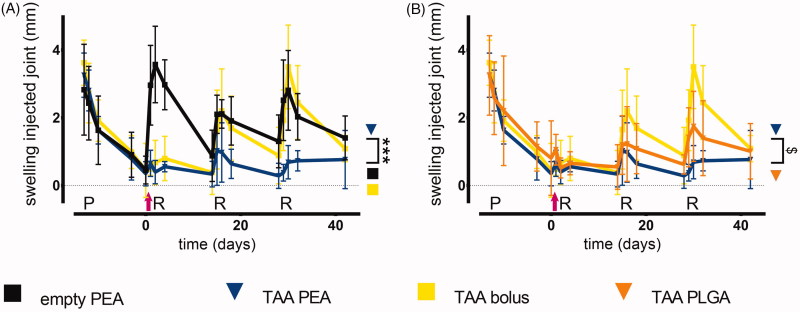
TAA-releasing PEA microspheres reduce joint swelling most efficaciously. Rats treated with a single intra-articular knee injection of (A) empty PEA microspheres *n* = 6 (black squares), TAA loaded PEA microspheres *n* = 5 (blue triangles) and TAA bolus suspension *n* = 6 (yellow squares), and (B) TAA loaded PEA microspheres *n* = 5 (blue triangles) benchmarked versus TAA bolus suspension *n* = 6 (yellow squares) and TAA loaded PLGA microspheres *n* = 6 (orange triangles). Swelling of the experimental knee joints is expressed as difference between joint thickness and baseline joint thickness. Pink arrow indicates time point of the intra-articular knee injection. P: priming, R: reactivation. Data are presented as mean ± SD, $*p* = .053 and ***p < .001.

**Figure 4.**
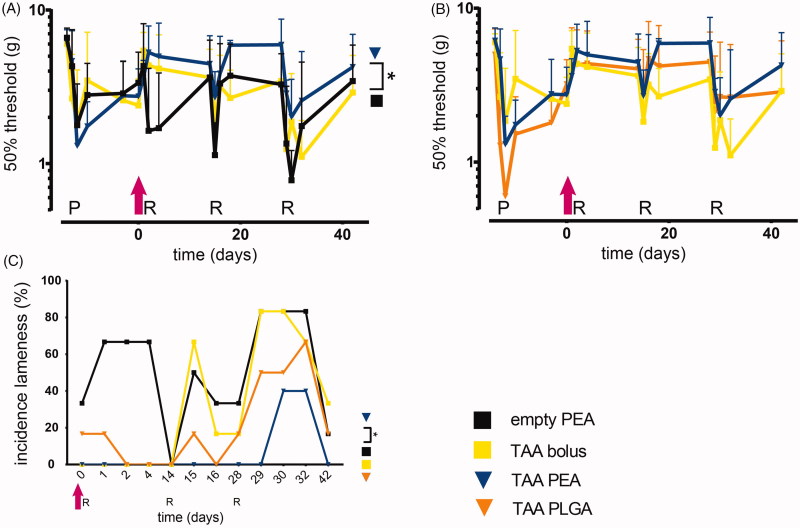
TAA release from PEA microspheres rescues synovitis-induced mechanical hypersensitivity and prevents lameness. As a measure for PGPS-induced referred mechanical hypersensitivity the 50% response threshold of the hind paws to von Frey hairs was determined in (A) animals treated with a single intraarticular knee injection (pink arrow) of empty PEA microspheres *n* = 6 (black squares), TAA loaded PEA microspheres *n* = 5 (blue triangles) and TAA bolus suspension *n* = 6 (yellow squares) or (B) animals treated with a single intra-articular knee injection of TAA loaded PEA microspheres *n* = 5 (blue triangles) benchmarked versus TAA bolus suspension *n* = 6 (yellow squares) and TAA loaded PLGA microspheres *n* = 6 (orange triangles). Data are presented as mean ± SD, *p < .05. (C) The incidence of lameness during the course of PGPS-induced synovitis was measured of animals treated with empty PEA microspheres *n* = 6, TAA loaded PEA microspheres *n* = 5, TAA bolus suspension *n* = 6, and TAA loaded PLGA microspheres *n* = 6. Statistical analysis was performed on the cumulative lameness scores. Significance was assumed at *p* < .0125, indicated by an asterisk. P: priming, R: reactivation.

**Figure 5.**
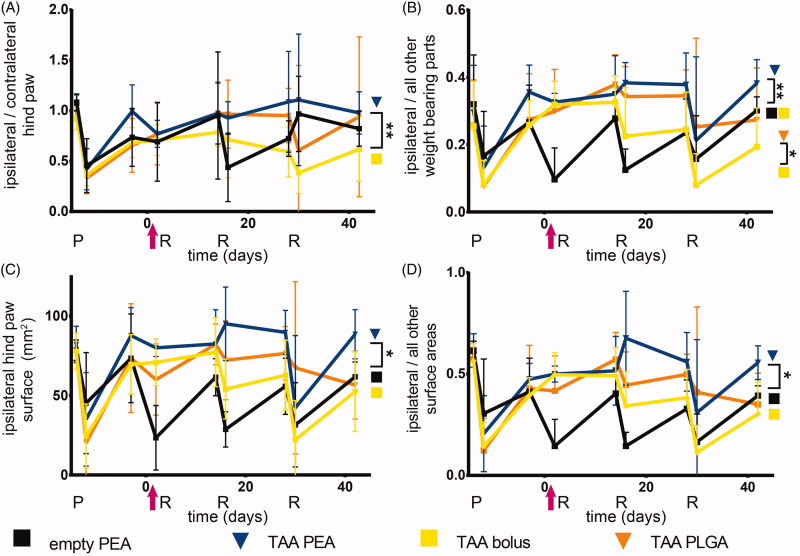
TAA release from PEA microspheres attenuated loss of weight bearing of the affected paw. Relative weight distributions and weight bearing surface area of animals were determined during the course of PGPSinduced synovitis. Rats were treated with a single intra-articular knee injection (pink arrow) of empty PEA microspheres *n* = 6 (black squares), TAA loaded PEA microspheres *n* = 5 (blue triangles), TAA bolus suspension *n* = 6 (yellow squares) or TAA loaded PLGA microspheres *n* = 6 (orange triangles). (A) Weight-bearing ratio of the affected paw (ipsilateral hind paw) compared to the contralateral hind paw. (B) Weight-bearing ratio of the affected paw (ipsilateral hind paw) compared to the total weight bearing of the nonaffected parts (front paws, contralateral paw, tail). (C) Weight-bearing surface area of the affected paw (ipsilateral hind paw) is depicted. (D) Surface area ratio of the affected paw (ipsilateral hind paw) compared to the total weight bearing of the non-affected parts (front paws, contralateral paw, tail) is displayed. P: priming, R: reactivation. Data are presented as mean ± SD, *p < .05, **p < .01.

PGPS priming and reactivations induced temporal inflammatory responses with remission 14 days post-injection in rats injected with empty PEA (Figure 3(A)). PGPS injections induced episodes of referred mechanical hypersensitivity that peaked at 4 days after reactivation and then diminished over time before the following reactivation. These episodes coincided with visual and objective measurements of lameness (Figure 4, an example of lame animal in Supplementary Video 1). With subsequent reactivation-induced synovitis flares, the magnitude of mechanical hypersensitivity appeared to increase compared to the previous flare in vehicle control-treated animals (Figure 4(A)). Synovitis flare-up reactivations also induced non-evoked pain behaviors, reflected by the reduced weight bearing and strong reductions in the loading surface of the affected hind paw (Figure 5). Joint swelling and pain-like behaviors did not completely return to baseline before reactivation, similar to what was observed in a previous study (Kumar et al., 2015). Furthermore, a few animals showed lameness in combination with swelling of both hind paws, leading to administration of s.c. carprofen as rescue medication (total interventions; empty PEA - 16, TAA bolus - 5, PEA TAA – 0 and PLGA TAA - 1). One rat receiving PEA microparticles loaded with TAA was euthanized before the end of the study (day 16) as it reached the humane endpoint of the study due to polyarthritis without improvement upon additional pain medication.

Joint swelling was also observed transiently after PGPS-induced reactivations in all contralateral knee joints, regardless of treatment (Supplementary Figure 1). Similarly, mechanical thresholds reduced in the contralateral joints with subsequent reactivations in control animals (Supplementary Figure 2).

**Figure 6.**
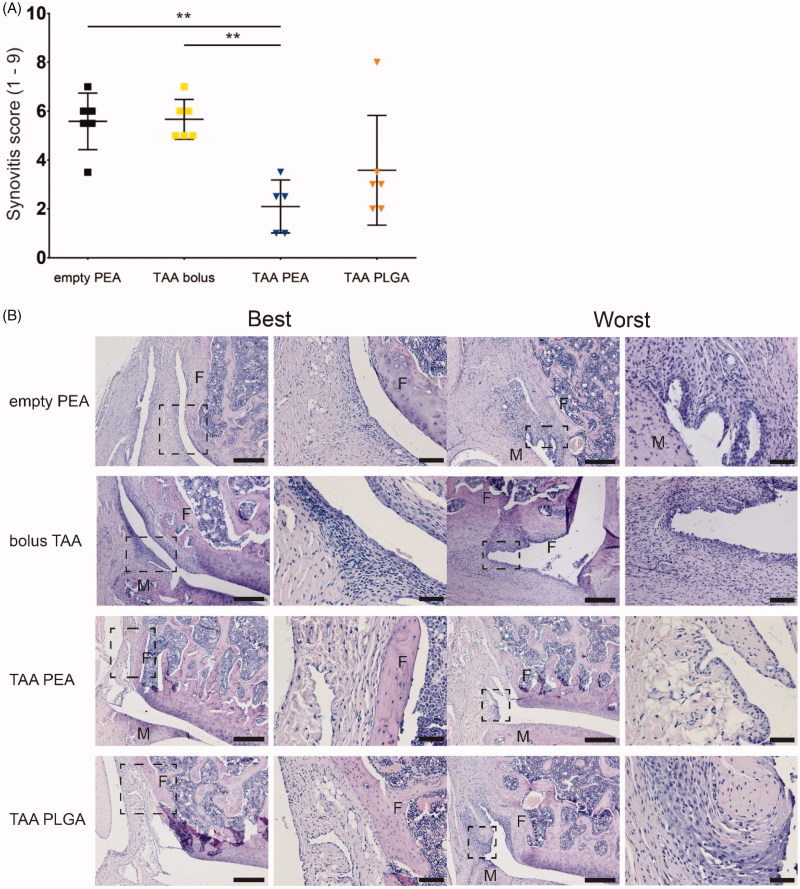
Single injection of TAA released from PEA microspheres reduced synovitis at day 42. (A) Extend of synovitis was quantified using the Krenn score of the knee joint of rats treated with an intra-articular knee injection of empty PEA microspheres *n* = 6 (black squares), TAA bolus suspension *n* = 6 (yellow squares), TAA loaded PEA microspheres *n* = 5 (blue triangles), or TAA loaded PLGA microspheres *n* = 6 (orange triangles). Data were presented as mean ± SD and significance was found when *p < .05, double asterisks indicate *p* < .01. F: femur, M: meniscus. (B) Histological representative images of the best and worst outcome of the Krenn scores of every experimental group.

Histopathologically, PGPS injections induced extensive activation of synovial stroma, synovial lining hyperplasia and immune cell infiltration (Figure 6) while only very mild cartilage degeneration was observed. None of the contralateral joints showed synovitis or cartilage degeneration (data not shown) and bone changes were absent on micro-CT in any of the joints at 42 days follow up (data not shown).

### Anti-inflammatory and analgesic capacities of TAA, released by microspheres

#### Swelling

A single TAA bolus administration inhibited swelling after the first reactivation, while the inhibitory effect on swelling seemed to decline with subsequent inflammatory flare-ups (Figure 3(A)). Prolonged exposure of TAA by PLGA microspheres reduced joint swelling over time, however, the reduction in joint swelling was not statistically different compared to TAA bolus injected joints (Figure 3(B)). In contrast, TAA-loaded PEA microspheres reduced swelling significantly more compared to TAA bolus injection over the total 42 days of follow up (Figure 3(A), *p* < .001). TAA-loaded PEA microspheres tended to reduce joint swelling more effectively than TAA-loaded PLGA microspheres (Figure 3(B), *p* = .053).

### Pain-like behavior

#### Lameness

A single TAA bolus injection could not prevent lameness induced by repeated PGPS injections (Figure 4(C)). Rats injected with TAA-loaded PLGA microspheres were less frequently lame after the first and second reactivation, but this effect was lost after the third reactivation. Rats treated with a single injection of TAA-loaded PEA microspheres displayed significantly less frequently lameness compared to TAA-loaded PLGA- and TAA bolus-treated rats over the complete study period (Figure 4(C); *p* < .0125).

#### Referred mechanical hypersensitivity

TAA bolus administration reduced the first reactivation-induced hypersensitivity, but the inhibition of mechanical hypersensitivity was lost with subsequent reactivation-induced flares (Figure 4(A)). TAA-loaded PLGA microspheres appeared to reduce reactivation-induced mechanical hypersensitivity up to the second reactivation compared to TAA- bolus treated rats, but no statistically significant difference was found (Figure 4(B)). Prolonged exposure of TAA-loaded PEA microspheres reduced flare-induced mechanical hypersensitivity (Figure 4(A); *p* < .05), although no statistical significant difference was found compared to TAA bolus administration. After the third reactivation at day 28, the analgesic effect of TAA-loaded PEA and PLGA microspheres was diminished. Although at most time points, the magnitude of hypersensitivity reduction appeared higher for the PEA platform, difference between the two microsphere platforms was not statistically significant (Figure 4(B)).

#### Dynamic weight bearing analysis

The reduced weight bearing of the ipsilateral affected paw compared to the contralateral paw of TAA bolus-treated rats was partially normalized, but after the second reactivation no beneficial effects of the TAA bolus were observed (Figure 5(A)). TAA-loaded PLGA microspheres normalized the weight bearing until the third reactivation, although no statistical differences with the effects of TAA bolus were found. TAA release by PEA microspheres normalized the weight bearing of the ipsilateral affected paw for the complete study period, compared to animals receiving TAA bolus injections (Figure 5(A), *p* < .01). No statistically significant difference was observed between microsphere treatments.

Weight-bearing of the affected paw compared to all other weight-bearing parts was normalized in animals after the first reactivation receiving TAA bolus and declined gradually after the second reactivation (Figure 5(B)). TAA-loaded PLGA and PEA microspheres normalized the weight bearing of the affected paw compared to all other weight-bearing parts compared to TAA bolus-treated animals (Figure 5(B), *p* < .05). Weight distribution of animals receiving TAA-loaded microspheres remained predominantly unchanged the first 28 days, without any clear effect between the two microsphere platforms.

In rats receiving TAA bolus suspension the loading surface area of the affected hind paw did not change after the first reactivation. The loading surface decreased with subsequent reactivations, indicating the analgesic effect of TAA bolus on paw eversion as consequence of pain was lost (Figure 5(C)). The arthritis-induced reduction in loading surface area was attenuated by IA injection with TAA-loaded PLGA microspheres after the first two reactivations, although no statistical differences were observed compared to TAA bolus-treated rats. The loading surface area of the affected hind paw remained unchanged in TAA-loaded PEA treated animals, compared to TAA bolus treated animals (Figure 5(C), *p* < .05). After the third reactivation, affected paw loading surface area of animals treated with TAA-loaded PEA microspheres returned to the levels of control animals. No significant differences in paw surface area reduction were observed between TAA-loaded PEA microspheres and PLGA microspheres.

The loading surface area of the affected hind paw compared to all other loading parts in animals receiving TAA bolus suspension was not affected after the first reactivation, indicating the absence of paw eversion as consequence of pain, and gradually decreased over time (Figure 5(D)). The loading surface area of the affected hind paw compared to all other loading parts remained unchanged in rats treated with TAA released by PLGA microspheres as well, up to two reactivations and thereafter decreased, although no statistical differences were observed compared to TAA bolus. Animals treated with TAA-loaded PEA microspheres did not unload the affected hind paw to redistribute the surface area to other parts, compared to TAA bolus animals (Figure 5(D), *p* < .05), up to the third reactivation. No significant differences in affected paw surface area reduction compared to other loading parts was observed between TAA-loaded PEA and PLGA microspheres.

### Histopathology

Histopathological assessment was performed on both knee joints to determine the extent of synovitis *post mortem* using the Krenn score and cartilage degeneration using the Mankin score. Knee joints of animals treated with either empty PEA or bolus TAA contained extensive activation of synovial stroma, enlarged synovial lining, and immune cell infiltration in the synovium (Figure 6). Although moderate synovial activation and slight cell infiltration were found in TAA-loaded PLGA microsphere treated joints, synovitis was not significantly suppressed, compared to TAA bolus-treated joint (Figure 6(A,B)). Sustained TAA release by the PEA microsphere platform suppressed synovitis induced by PGPS injections significantly compared to TAA bolus or PEA control injections (Figure 6(A); *p* < .01) at 42 days follow up. No significant differences were found between any of the experimental groups in the extent of cartilage degeneration (Supplementary Figure 1).

## Discussion

Targeting synovial inflammation in osteoarthritis has the potential to reduce clinical symptoms such as joint swelling and pain (Benito et al., 2005; Ene et al., 2015). To suppress pain in inflammatory joint disease, intra-articular biomaterial-based release of an anti-inflammatory drug can prolong retention of the drug in the joint space and thereby prolong the anti-inflammatory effects. However, release kinetics and thus anti-inflammatory duration are dependent on biomaterial properties (Tsitlanadze et al., 2004; Doty et al., 2017). To this end, we tested the capacity of the enzyme-degradable PEA biomaterial as a microsphere platform for prolonged release of the corticosteroid TAA in an acute arthritis model and compared it with a commercially used bulk eroding PLGA platform and a standard formulation of TAA for clinical care. In the model of PGPS-induced inflammatory synovitis (Esser et al., 1985; Kumar et al., 2015), a single TAA bolus injection of Kenalog® relieved inflammation and pain after the first flare-up reactivation but thereafter with subsequent reactivations this relief of pain and inflammation gradually was lost. Prolonged exposure of TAA released by PEA microspheres inhibited pain-like behavior more compared to bolus TAA exposure over the complete study period consisting of three reactivations, while PLGA microspheres were only effective during the first two flare-up reactivations. Swelling in joints injected with TAA-loaded PLGA microspheres seemed more pronounced compared to knee joints injected with TAA-loaded PEA microspheres. However, despite the consistent trends increased the efficiency of PEA-mediated delivery, no statistically significant differences between the two microsphere platforms were found for any of these parameters. Only lameness was more frequently observed in animals treated with TAA-loaded PLGA microspheres and histopathological hallmarks of synovitis of rats injected with TAA releasing PEA microspheres were less severe.

Extending drug exposure locally using controlled release platforms is a recently introduced therapeutic strategy in OA research (Kavanaugh et al., 2016), with one PLGA-based product on the market (Kaufman, 2017). The observed reduction in pain-like behavior in the current study is in line with previous studies using PLGA for TAA delivery (Horisawa et al., 2002; Kumar et al., 2015). However, in contrast to the current study, TAA-loaded PLGA microspheres inhibited limping more efficiently than TAA bolus in the same model, predominantly the first 28 days after delivery (Kumar et al., 2015). In the current study, the gradual decline in analgesic effect was less pronounced in TAA PEA-treated animals compared to the PLGA-based TAA delivery. However, these effects were not statistically significant, most likely due to the high variance in the pain read-out parameters and the limited power for detection of differences between the platforms. Nevertheless, PEA-mediated TAA delivery showed a significant difference compared to bolus administration for several pain parameters, whereas almost none differed when the PLGA platform was compared to bolus, indirectly suggesting PEA-mediated delivery was more effective than PLGA. Notably, the increased number of readout parameters in this study also increase the chances of finding statistically significant differences (Parsons et al., 2018).

Altogether these results indicate that TAA release from PEA microspheres results in effective and sustained analgesia. The mechanism behind the suggested difference in effects of the platforms might be the different degradation properties of the polymers *in vivo* (Peters et al., 2017). Delivery from PLGA microspheres is dependent on diffusion and bulk erosion, whilst PEA degradation is driven by serine protease activity. Serine protease activity is mainly observed during inflammation in OA (Muley et al., 2017), which activity is reduced when inflammation is downregulated by released TAA, creating a feedback loop on the degradation of the TAA-releasing PEA platform (Janssen et al., 2016), accounting for a prolonged analgesia *in vivo*.

In this model of acute arthritis, more akin to rheumatoid arthritis, we observed that the contralateral joints also showed swelling and mechanical hypersensitivity after systemic reactivation. Strikingly, however, none of the contralateral knee joints showed significant synovitis *post mortem*, indicating that systemic reactivation induces some signs of inflammation (dolor, tumor) but not a full-blown synovitis. Another possibility is that the unilateral inflammation generated contralateral hypersensitivity due to plasticity of the central nervous system, including the spinal cord (Rijsdijk et al., 2017). However, pain symptoms are often observed before signs of inflammation are present in a joint developing RA, and increased pain sensitivity of non-inflamed sites has been observed in RA patients (Lee et al., 2009), while synovitis and pain do not always correlate (Forslind & Svensson, 2016). This discrepancy between inflammation and pain was also found in knee OA patients, where pain intensity poorly correlated with the degree of inflammation (Siebuhr et al., 2014; Petersen et al., 2016; Swaminathan et al., 2017; Riis et al., 2017). This may be related to the observation that both in RA and OA, pain can arise from pain signals by the peripheral (joint), spinal and supraspinal pain pathways (Zhang & Lee, 2018; O'Neill & Felson, 2018). However, the pathobiology of these joint diseases is very different and hence effects on pain-like behavior in this arthritis model should be interpreted with caution when extrapolating towards the treatment of OA pain. Still, TAA is used to alleviate OA joint pain and the controlled release of TAA from PLGA microspheres for OA joint pain, evaluated in the same arthritis model, is now launched as a commercially available product (Kaufman, 2017). Using this model, our data clearly indicate that biomaterial-based delivery of TAA has the potency towards long term reduction of inflammation and joint pain in OA.

The importance of the interaction between the synovium and cartilage in OA has been stressed before (Lindblad & Hedfors, 1987; Sellam & Berenbaum, 2010; Beekhuizen et al., 2011) with synovitis accelerating cartilage destruction and disease progression (Sellam & Berenbaum, 2010). However, in this study only mild cartilage degeneration was observed, with no evident differences noted between joints treated with bolus or slow released TAA. These results are in line with a previous study on the effects of controlled release of TAA in the same model, showing only limited cartilage damage, with slight improvements after TAA exposure (Kumar et al., 2015). Possibly the periodic synovitis in this model allows for tissue recovery in between. However, the evidence is also accumulating that rather than mediating cartilage breakdown, ECM breakdown products propagate synovial inflammation in OA, possibly due to Toll-like receptor signaling in the synovium (Scanzello & Goldring, 2012; Wight et al., 2014). Hence the value of corticosteroid therapy for preserving cartilage integrity may be limited (Raynauld et al., 2003; Yavuz et al., 2012) in OA.

## Conclusion

To conclude, a PEA microsphere platform for TAA delivery reduced swelling, pain, lameness, and synovial inflammation in a model of acute arthritis, and was more efficient in reducing lameness and histological synovitis compared to PLGA-based delivery as reference drug delivery system. Future studies should consider assessing higher dosages and/or OA models involving cartilage damage to provide a better understanding of the effect of prolonged TAA exposure on all joint tissues.

## Supplementary Material

NC3Rs_ARRIVE_Guidelines_Checklist_filled.pdf

sup_figure_2.docx

sup_figure_1.docx

movie_lameness.mp4
