# Supplementary material for: Intra-articular injection of triamcinolone acetonide releasing biomaterial microspheres inhibits pain and inflammation in an acute arthritis model
Source: Drug Deliv. 2019 Mar 7;26(1):226–36. doi: 10.1080/10717544.2019.1568625 (PMC6407600; doi:10.1080/10717544.2019.1568625)
Supplement: sup_figure_1.docx [file IDRD_A_1568625_SM5249.docx]

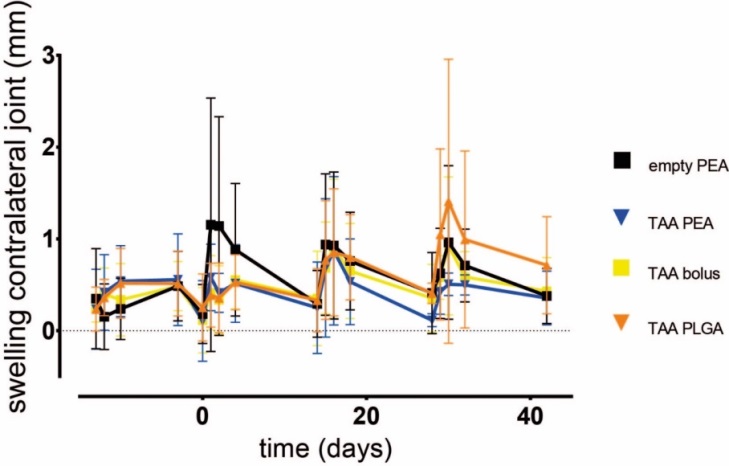


**Supplementary figure 1.** Swelling of the contralateral knee joints was calculated (joint thickness – baseline joint thickness). Intra-articular knee injection of treatments was administered at day 0; empty PEA microspheres *n=6* (black squares), TAA loaded PEA microspheres *n=5* (blue triangles), TAA bolus suspension *n=6* (yellow squares) and TAA loaded PLGA microspheres *n=6* (orange triangles). Data are presented as mean ± SD.
